# Neurodevelopmental disorders and gender dysphoria: a fertile relationship?

**DOI:** 10.1192/j.eurpsy.2022.2085

**Published:** 2022-09-01

**Authors:** I. Soares Da Costa, M. Mota

**Affiliations:** 1 CHUSJ, Psychiatry, Porto, Portugal; 2 Centro Hospitalar Universitário São João, Psychiatry And Mental Health Department, Porto, Portugal

**Keywords:** psychiatry, sexual medicine, Neurodevelopmental disorders, Gender Dysphoria

## Abstract

**Introduction:**

Development of gender identity is a complicated process. During this process it is thought that many factors play a role. Gender dysphoria is a condition where there is a mismatch between the assigned gender at birth and gender identity. Although scarce, literature shows that compared to cisgender individuals, transgender and gender-diverse individuals have higher rates of autism, other neurodevelopmental and psychiatric diagnoses.

**Objectives:**

To describe posible relations and overlap between gender dysphoria and neurodevelopmental disorders.

**Methods:**

Literature search in Pubmed and other similar platforms. Articles considered relevant under this theme were included.

**Results:**

Autism spectrum disorders (ASD) and attention-deficit hyperactivity disorder (ADHD) can compromise health and may be more prevalent amongst individuals with gender dysphoria (GD). Symptoms such as attention difficulties, deficits in communication and social skills, obsessional interests, and stereotyped behaviour can significantly impact assessment of GD and the appropriate clinical care. With some overlapping symptoms, the potential for misdiagnosis is possible. Data about prevalence of this conditions in transgender community is of low quality, but ASD is more prevalent, ranging from 6-26%.

**Conclusions:**

Studies demosntrate that neurodevelopmental disorders and other psychiatric conditions are more common in transgender and gender-diverse individuals. It is important that future studies focus on exploring the mental health outcomes of neurodevelopmental-trans individuals.

**Disclosure:**

No significant relationships.

